# Centipede grass exerts anti-adipogenic activity through inhibition of C/EBPβ, C/EBPα, and PPARγ expression and the AKT signaling pathway in 3T3-L1 adipocytes

**DOI:** 10.1186/1472-6882-12-230

**Published:** 2012-11-26

**Authors:** Hyoung Joon Park, Byung Yeoup Chung, Min-Kwon Lee, Yuno Song, Seung Sik Lee, Gyo Moon Chu, Suk-Nam Kang, Young Min Song, Gon-Sup Kim, Jae-Hyeon Cho

**Affiliations:** 1 Institute of Life Science, College of Veterinary Medicine, Gyeongsang National University, Jinju, Korea; 2 Advanced Radiation Technology Institute, Korea Atomic Energy Institute, Jeongeup, Korea; 3 Dept. of Animal Science & Biotechnology, Gyeongnam National University of Science and Technology, Jinju, Korea; 4 Department of Anatomy and Developmental Biology, College of Veterinary Medicine, Gyeongsang National University, 900 Gajwa-dong, Jinju-city 660-701, Korea

## Abstract

**Background:**

Centipede grass (CG) originates from China and South America and is reported to contain several C-glycosyl flavones and phenolic constituents, including maysin and luteolin derivatives. This study aimed to investigate, for the first time, the antiobesity activity of CG and its potential molecular mechanism in 3T3-L1 cells.

**Methods:**

To study the effect of CG on adipogenesis, differentiating 3T3-L1 cells were treated every day with CG at various concentrations (0–100 μg/ml) for six days. Oil-red O staining and triglyceride content assay were performed to determine the lipid accumulation in 3T3-L1 cells. The expression of mRNAs or proteins associated with adipogenesis was measured using RT-PCR and Western blotting analysis. We examined the effect of CG on level of phosphorylated Akt in 3T3-L1 cells treated with CG at various concentration s during adipocyte differentiation.

**Results:**

Differentiation was investigated with an Oil-red O staining assay using CG-treated 3T3-L1 adipocytes. We found that CG suppressed lipid droplet formation and adipocyte differentiation in 3T3-L1 cells in a dose-dependent manner. Treatment of the 3T3-L1 adipocytes with CG resulted in an attenuation of the expression of adipogenesis-related factors and lipid metabolic genes. The expression of C/EBPα and PPARγ, the central transcriptional regulators of adipogenesis, was decreased by the treatment with CG. The expression of genes involved in lipid metabolism, aP2 were significantly inhibited following the CG treatment. Moreover, the CG treatment down-regulated the phosphorylation levels of Akt and GSK3β.

**Conclusions:**

Taken collectively, these data indicated that CG exerts antiadipogenic activity by inhibiting the expression of C/EBPβ, C/EBPα, and PPARγ and the Akt signaling pathway in 3T3-L1 adipocytes.

## Background

Obesity is an important issue in the field of preventive medicine and public health because it is considered to be a risk factor associated with the development of multiple diseases, including heart disease, hypertension, and diabetes [1]. Recently, obesity has become the leading metabolic disease and is a significant problem owing to the increased risk of premature death. Adipocytes play a critical role in regulating lipid metabolism and energy balance and are associated with adipose tissue mass and obesity. Indeed, obesity is induced by the hypertrophy of adipocytes and the generation of new adipocytes from precursor cells [2]. As lipid accumulation reflects the process of adipogenesis and the programmed differentiation of preadipocytes involves several stages related to obesity [3], many studies have aimed to reduce obesity by focusing on decreasing preadipocyte differentiation and proliferation, inhibiting lipogenesis, and increasing lipolysis.

Adipocyte differentiation is a complex process that is regulated by various transcription factors and adipogenesis-related genes. The initial events are orchestrated by several transcriptional factors, CCAAT/enhancer binding protein-β (C/EBPβ), peroxisome proliferator-activated receptor-γ (PPARγ) and CCAAT/enhancer binding protein-α (C/EBPα) [4]. C/EBPβ is expressed in the early stage of adipocyte differentiation and activates the transcription of C/EBPα and PPARγ, transcription factors that activate the expression of the adipocyte genes that give rise to the adipocyte phenotype [4,5]. The expression of C/EBPα and PPARγ is also associated with terminal differentiation, and these factors act in concert to generate fully mature adipocytes by their subsequent transactivation of adipocyte-specific genes [6]. During adipogenesis, PPARγ and C/EBPα activate the expression of lipid-metabolizing enzymes, such as fatty acid binding protein 4 (aP2), lipoprotein lipase (LPL), and fatty acid synthetase (FAS) [6,7].

The serine/threonine kinase Akt plays an essential role in adipocyte differentiation. Mouse embryonic fibroblasts lacking Akt have impaired ability to differentiate into mature adipocytes [8], and an RNAi-mediated decrease of Akt is found to block the differentiation of 3T3-L1 cells [9]. Moreover, the overexpression of constitutively active Akt results in increased glucose uptake and adipocyte differentiation in 3T3-L1 adipocytes [9]. Akt phosphorylates and regulates a large number of substrates involved in a diverse array of biological process [10], and it is essential for the induction of PPARγ expression [11]. GSK3β is a critical downstream signaling protein for the phosphoinositide 3-kinase (PI3K)/Akt pathway. Insulin signaling activates Akt through PI3K and induces serine/threonine phosphorylation of the downstream target, GSK3β, which phosphorylates C/EBPβ, C/EBPα, and glycogen synthase (GS) [12,13].

Centipede grass (CG) (Eremochloa ophiuroides) is native to China and Southeast Asia, and it is now one of the most popular lawn grasses in South America [14]. Wiseman et al. showed that CG has a several C-glycosyl flavones and phenolic constituents [15], and a recent study has reported that the methanolic extracts from the leaves of CG exhibited an inhibitory effect against pancreatic lipase [16].

In the present study, the effect of CG on the adipocyte differentiation of 3T3-L1 cells was investigated by measuring lipid accumulation and the expression levels of adipocyte marker genes and their target genes. Moreover, to understand the specific mechanisms of these effects, we examined whether Akt and GSK3β activation are critical for the anti-adipogenic functions of CG.

## Methods

### Preparation of centipede grass

The extract of Centipede grass (CG) (Eremochloa ophiuroides) was supplied by Korea Atomic Energy Institute (KAERI). CG extract contains Luteolin-6-C-boivinopyranoside, orientin, isoorientin, derhamnosylmaysin, isoorientin 2-O-α-L-rhamnoside, and luteolin and the extraction procedure was previously described [16].

### Cell viability and cytotoxicity

The cytotoxic effects of CG, and its effects on cell viability, were determined in LDH and MTT assays. Cells were seeded at a density of 1 x 10^5^ cells/well into 24 well plates. After 24 h, they were treated with CG for 4 day or 6 day. MTT was added to each well, and the plates incubated for 4 h at 37°C. The liquid in the plate was removed, and dimethyl sulfoxide (DMSO) added to dissolve the MTT-formazan complex formed. The optical density was measured at 540 nm. The effect of CG on cell viability was evaluated as absorbance relative to that of control cultures. The cytotoxicity effect of CG was measured using an LDH cytotoxicity detection kit (Roche Applied Science, USA). LDH activity was measured, according to the manufacturer’s protocol, in culture supernatants and cell lysates to evaluate cytotoxicity.

### Cell culture and differentiation

Mouse 3T3-L1 preadipocytes were grown in Dulbecco’s modified eagle medium (DMEM) high glucose with 10% calf serum at 37°C in a humidified atmosphere of 5% CO_2_. After 3T3-L1 cells reached confluency, the cells were incubated in differentiation/induction medium (DMII) containing 167 nM insulin, 0.5 mM 3-isobutyl-1-methylxanthine, 100 μM indomethacin, and 0.25 μM dexamethasone in DMEM containing 10% FBS. The differentiation/induction medium was changed every 2 days. To examine the effects of CG on the differentiation of preadipocytes to adipocytes, cells were cultured in DMII differentiation medium in the presence of various concentrations (10 μg/ml or 100 μg/ml) of centipede grass. The differentiated cells were used after 4 or 6 days of initiating the differentiation.

### Oil-red O staining

After the induction of differentiation, cells were stained with Oil-red O. Cells were treated either with CG extracts (10 μg/ml or 100 μg/ml) or vehicle in the differentiation medium for day 0–6 of adipogenesis. On day 4 or 6, cells were stained with Oil-red O. For Oil-red O staining, cells were washed gently with PBS, and stained with filtered Oil-red O solution for 30min. After staining the lipid droplets red, the Oil-red O staining solution was removed and the plates were rinsed with water and dried. The stained lipid droplets were viewed on an Olympus microscope (Tokyo, Japan).

### Triglyceride content assay

Cellular triglyceride contents were measured using a commercial triglyceride assay kit (Sigma-Aldrich, MO, USA) according to the manufacturer’s instructions. Adipocytes differentiated for 4 or 6 days were treated with the CG at concentrations of 0, 10 and 100 μg/ml in 6-well plates. To analyze the content of cellular triglycerides, cells were washed with PBS and then scraped into 200 μl PBS and homogenized by sonication for 1min. The lysates were assayed for total triglycerides by using the assay kits.

### RNA preparation and RT-PCR

Cellular RNA was isolated from 3T3-L1 adipocytes using Trizol reagent (Invitrogen, USA) according to the manufacturer’s instructions. One microgram of total RNA was subjected to first strand cDNA synthesis with oligo (deoxythymidine) primers and Superscript II reverse transcriptase (invitrogen, CA, USA). Gene expression was normalized using β-actin as a reference gene. Data were analyzed using Opticon Monitor software (Bio-Rad). The target cDNA was amplified using the following sense and antisense primers: sense 5′-GACTACGCAACACACGTGTAACT-3′ and antisense 5′-CAAAACCAAAAACATCAACAACCC-3′ for C/EBPβ; sense 5′-TTT-TCA-AGG-GTGCCA-GTT-TC-3′ and antisense 5′-AAT-CCT-TGG-CCCTCT-GAG-AT-3′ for PPARγ; sense 5′-TGA-AGA-GGTCGG-CGA-AGA-GTT-CG-3′ and antisense 5′-GGC-GGT-CAT-TGT-CAC-TGG-TCA-AC-3^′^ for C/EBPα; Control detection of β-actin was performed with sense (5′- GACAACGGCTCCGGCATGTGCAAAG-3′) and antisense (5′-TTCACGGTTGGCCTTAGGGTTCAG-3′) primers under the same conditions.

### Western blot analysis

Western blotting was performed according to standard procedures. Briefly, cells were washed with ice-cold PBS, and lysed in lysis buffer (50 mM Tris–HCl (pH 8.0), 0.4% Nonidet P-40, 120 mM NaCl, 1.5 mM MgCl_2_, 0.1% SDS, 2 mM phenylmethylsulfonyl fluoride, 80 μg/ml leupeptin, 3 mM NaF and 1 mM DTT). After cell debris was removed by centrifugation, lysate protein concentrations were determined using Bio-Rad protein Assay Reagent (Bio-Rad Laboratories, USA). Cell lysates were then subjected to electrophoresis on 10-15% SDS-polyacrylamide gels containing SDS, and transferred onto a polyvinylidene fluoride membrane (Amersham Pharmacia, UK). PPARγ, C/EBPβ, C/EBPα, aP2, Akt, and GSK3β antibody were from cell signaling and the monoclonal β-actin antibody was from Chemicon. HRP-labeled mouse anti-rabbit IgG were from Jackson ImmunoResearch. The Chemiluminescence kit was from Pierce (Rockford, IL). After incubation with horseradish-peroxidase-conjugated secondary antibody at room temperature, immunoreactive proteins were detected using a chemiluminescent ECL assay kit (Amersham Pharmacia, UK) according to the manufacturer’s instructions.

### Statistical analysis

Data are expressed as means ± SD. Comparison between groups made by ANOVA variance analysis, and significant was analyzed by Ducan’s multiple range tests. Differences of p < 0.05 were considered to be statistically significant.

## Results

### Effect of CG on the adipocyte differentiation of 3T3-L1 preadipocytes

**Figure 1:**
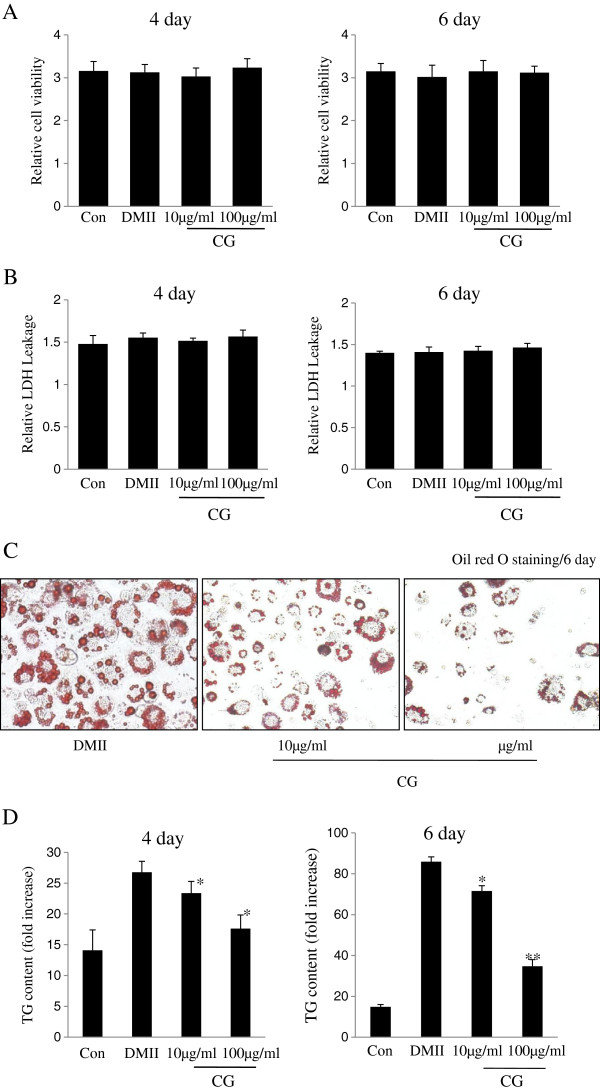
**Effect of Centipede grass on 3T3-L1 preadipocyte differentiation.** 3T3-L1 preadipocytes were induced to differentiate as described in Materials and Methods. At day 4 or 6, cell viability and cytotoxicity were assessed in MTT and LDH assays. (**A**) 3T3-L1 preadipocytes was incubated with DMII media for 4 or 6 days and treated with different concentration of CG every day. Con, 3T3-L1 preadipocytes; DMII, fully differentiated adipocytes (0.5 mM 3-isobutyl-1-methylxanthine, 100 μM indomethason, 0.25 μM dexamethasone and 167 nM insulin); 10 μg/ml, fully differentiated adipocytes (DMII + 10 μg/ml CG); 100 μg/ml, fully differentiated adipocytes (DMII + 100 μg/ml CG). Cell viability was determined by MTT assay. Results represent the mean ± SD of three independent experiments. (**B**) LDH assay was performed to determine cytotoxicity during differentiation. Results represent the mean ± SD of three independent experiments. (**C**) 3T3-L1 preadipocytes were differentiated into adipocytes in DMII medium contained with or without different concentration of CG for 6 days. On day 6, cells were stained with Oil-red O to visualize lipid accumulation. (**D**) CG reduced TG content during differentiation of 3T3-L1 cells. 3T3-L1 preadipocytes were differentiated in the absence or presence of CG for 4 or 6 days, and the lipid accumulation was measured by triglyceride assay. Data are mean ± SD values of at least three independent experiments. (*) p < 0.05, (**) P < 0.01 compared with the differentiated adipocytes (DMII).

The 3T3-L1 cells line, derived from mouse embryo fibroblasts, has been used as a model of adipogenic differentiation and insulin action. During differentiation, the cells undergo growth arrest and initiate differentiation that is manifested by gene expression and the morphological characteristics of mature adipocytes, such as the accumulation of lipid droplets. To assess any cytotoxic effects of CG, cell viability and cytotoxicity in 3T3-L1 adipocytes were evaluated using MTT and LDH assays. The 3T3-L1 cells were treated with different concentrations of CG (0, 10, or 100 μg/ml) with a DMII mixture for 4 or 6 days. At a concentration of 100 μg/ml, CG had no significant inhibitory effects on cell viability and did not cause cytotoxicity in the 3T3-L1 cells after 4 or 6 days of incubation, as determined by the MTT and LDH assays (Figure 1A, B). The 3T3-L1 preadipocytes were also treated at various concentrations (100, 200, and 300 μg/ml) for 6 days without differentiation, and CG did not show any cytotoxicity at up 200 μg/ml. Next, to determine the potential inhibitory effects of CG extracts on adipocyte differentiation, 3T3-L1 preadipocytes were differentiated in DMII alone or with DMII and CG (0, 10, or 100 μg/ml) during adipocyte differentiation for 6 days. The 3T3-L1 cells were fully differentiated by 6 days, and the accumulation of lipids was visualized with a microscopic inspection and Oil-red O staining. The treatment of CG resulted in a strong reduction of the lipid droplets in a dose-dependent manner in the 3T3-L1 adipocytes (Figure 1C). The cells treated with the DMII mixture plus 100 μg/ml of CG extract accumulated 65% of the intracellular triglyceride level contained in the DMII mixture-treated positive controls, as shown by the Oil-red O staining (Figure 1C). These results indicated that CG (100 μg/ml) inhibited lipid accumulation without exerting cytotoxicity during 3T3-L1 differentiation under DMII culture conditions. Next, to investigate the effect of CG on adipocyte differentiation, 3T3-L1 cells were treated with various concentrations of CG in the presence of DMII. As a major marker of adipogenesis, lipid accumulation was quantified by measuring the triglyceride content during 3T3-L1 differentiation. The triglyceride content of the cells increased during the 3T3-L1 differentiation for 6 days, and the addition of CG into the differentiation medium completely blocked the triglyceride accumulation (Figure 1D). The inhibitory effects of CG on the triglyceride accumulation during adipocyte differentiation were dose dependent and time dependent. These results indicated that CG exerts antiadipogenic activity in 3T3-L1 adipocytes.

### Effect of CG on the expression of adipogenic-specific genes during adipocyte differentiation

**Figure 2:**
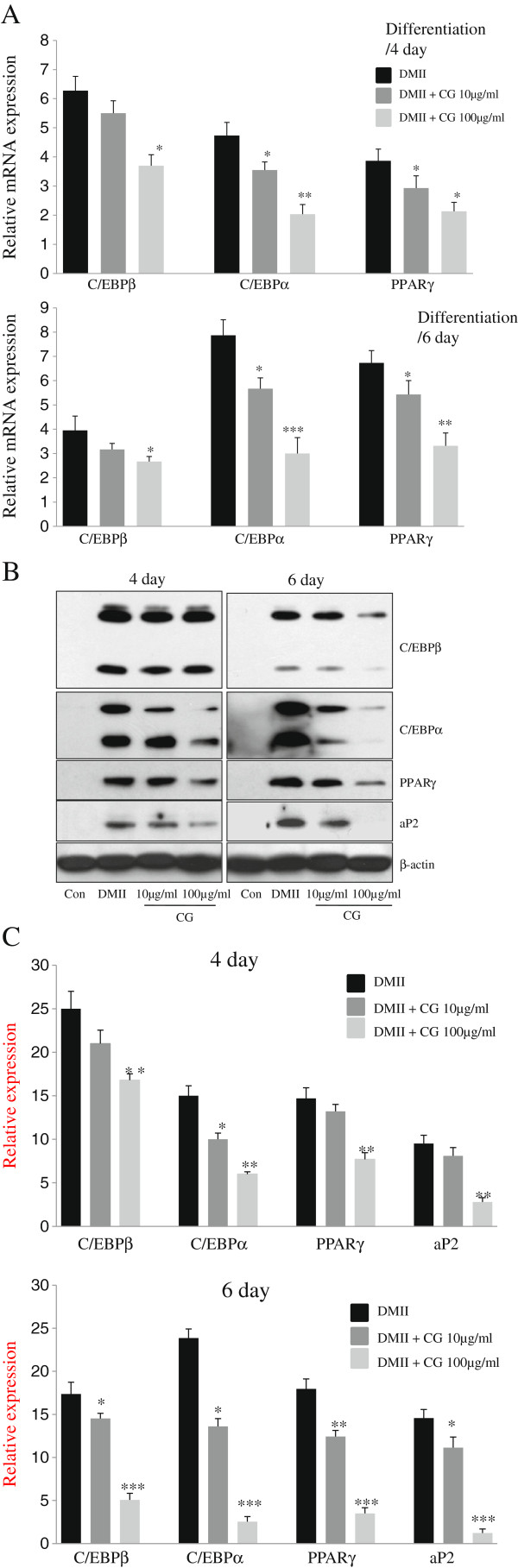
**Effect of CG on expression of adipogenesis-related genes.** 3T3-L1 preadipocytes were differentiated into adipocytes in DMII medium with the absence or presence of 10 μg/ml or 100 μg/ml CG for 4 or 6 days. Con, 3T3-L1 preadipocytes; DMII, fully differentiated adipocytes (0.5 mM 3-isobutyl-1-methylxanthine, 100 μM indomethason, 0.25 μM dexamethasone and 167 nM insulin); 10 μg/ml, fully differentiated adipocytes (DMII + 10 μg/ml CG); 100 μg/ml, fully differentiated adipocytes (DMII + 100 μg/ml CG). **(A)** CG inhibited the expression of adipocyte-specific transcription factors during differentiation. The gene expression was performed by RT-PCR and all gene expression was normalized using β-actin as reference gene. All experiments were performed in three independent experiments. (*) p < 0.05, (**) P < 0.01, (***) P < 0.001 versus the control (DMII) group at each gene expression. **(B)** CG attenuated the expression of adipogenesis-related genes in 3T3-L1 adipocytes. Total cell lysates were isolated from 3T3-L1 adipocytes at day 4 or day 6 after induction of differentiation. Immunoblotting analysis was performed as described in Methods. **(C)** The relative expression of adipogenesis-related genes after treating CG for 4 or 6 days. All gene expressions were normalized using β-actin as a reference gene. The data shown are representative of three independent experiments. (*) p < 0.05, (**) P < 0.01, (***) P < 0.001 versus the control (DMII) group at each gene expression.

Next, we investigated the influence of CG on the expression of several adipogenic genes. To determine whether the reduced accumulation of intracellular lipid droplets resulted from a CG extract-mediated alteration in the 3T3-L1 differentiation, the expression of the adipogenic-specific genes, C/EBPβ, PPARγ, and C/EBPα, were examined in the cells cultured in the presence or absence of CG (10 or 100 μg/ml) with adipogenic stimulation for 4 or 6 days. Our RT-PCR analysis revealed that the expression of C/EBPβ, C/EBPα, and PPARγ mRNA was induced in the adipocytes when the 3T3-L1 cells had become fully differentiated, whereas the expression of these genes was significantly decreased by CG (Figure 2A). CG treatment at 10 μg/ml or 100 μg/ml decreased the expression of C/EBPα mRNA by 28% or 59%, respectively, in the fully differentiated adipocytes, and the expression of PPARγ mRNA was decreased by approximately 21% or 55% compared to the fully differentiated cells (Figure 2A). To determine the protein expression patterns of adipogenic-specific genes during 3T3-L1 differentiation, we performed western blot analyses using antibodies against C/EBPβ, C/EBPα, and PPARγ. When CG was added with the DMII medium to the 3T3-L1 adipocytes, the expression of C/EBPβ, an early phase adipogenic transcription factor, was suppressed compared to the positive control cells, which were incubated with DMII only. We also determined the expression level of PPARγ and C/EBPα, which regulate adipogenesis and strictly associated with the initial appearance of lipid droplets during 3T3-L1 differentiation, respectively. Our results showed that the DMII mixture markedly increased the expression of C/EBPα and PPARγ, whereas the addition of CG to the 3T3-L1 cells significantly decreased the expression of C/EBPα and PPARγ protein at day 4 during the late stages of adipocyte differentiation (Figure 2B and C). To investigate whether the attenuated expression of PPARγ and C/EBPα further affected the activation of their target gene, aP2, we investigated the expression of aP2 under the same conditions. Treatment with CG significantly down-regulated aP2 expression compared to the fully differentiated 3T3-L1 adipocytes (Figure 2B and C).

### Effect of CG on the Akt pathway

**Figure 3:**
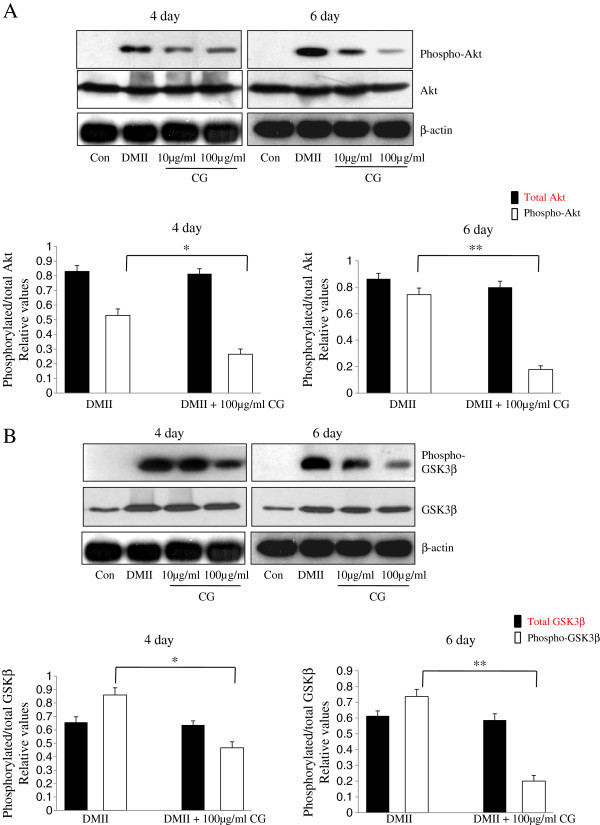
**Effect of CG on phosophorylation of Akt and GSK3β during 3T3-L1 differentiation.** 3T3-L1 preadipocytes were differentiated in the absence or in the presence of CG for 4 or 6 days. (**A**, **B**) 3T3-L1 adipocytes were treated with CG for indicated concentration and phosphorylation levels for Akt and GSK3β were determined by Western blotting analysis. The extent of phosphorylation was calculated by determining the amount of phosphorylated Akt relative to total Akt levels (DMII or DMII + 100 μg/ml CG). Data are mean ± SD values of at least three independent experiments. (*) p < 0.05, (**) P < 0.01.

To investigate the effect of CG on the regulation of the Akt pathway, we examined whether CG changed the phosphorylation levels of the downstream molecules of insulin signaling. The 3T3-L1 cells were treated with various concentrations of CG (0, 10, or 100 μg/ml) during differentiation, and, on day 4 or 6, the level of expression and phosphorylation of Akt and its substrate, GSK3β, a well-established cellular indicator of Akt activation, were examined. Lysates were collected and immunoblotted with phospho-independent Akt or GSK3β antibodies, phospho-Thr473 Akt (which recognizes Akt when Thr471 is phosphorylated), and phospho-Ser9 GSK3β (which recognizes GSK3β when Ser9 is phosphorylated). In the presence of DMII, the level of Akt phosphorylation (Thr-473) was highly increased, whereas treatment with CG decreased the level of the phosphorylated form of Akt (Figure 3A). The phosphorylation of GSK3β under the DMII mixture resulted in a robust increase in phospho-Ser9 immunoreactivity. In contrast to these findings, the treatment of 3T3-L1 adipocytes with 100 μg/ml CG resulted in a large decrease in the phosphorylation level of GSK3β (Figure 3B). These results demonstrated that CG treatment markedly inhibited the phosphorylation of Akt and GSK3β, leading to the inhibition of adipocyte differentiation by suppressing the Akt pathway.

## Discussion

Centipede grass has several C-glycosyl flavones, lueteolin 6-C-beta-D-boivinopyranoside and mayosin, which functions as a pancreatic lipase inhibitor [16]. In the present study, we carried out experiments aimed at determining the mechanism of the antiobesity activity of CG. The treatment of 3T3-L1 preadipocytes with CG inhibited their differentiation in a dose-dependent manner. CG treatment prevented the adipocyte differentiation by suppressing Akt activity, which sequentially attenuated the expression of C/EBPβ, C/EBPα, and PPARγ, key adipogenic transcription factors. The expression of aP2, terminal differentiation marker of adipocytes, was significantly inhibited by the treatment with CG.

It has been demonstrated that adipocyte differentiation and fat accumulation are associated with the development of obesity [2]. Increase in the adipocyte number and mass results from the massive adipocyte differentiation process, which generates mature adipocytes from preadipocytes [3]. One of the best-characterized and widely used in vitro models to study adipocyte differentiation has been the 3T3-L1 preadipocyte cell line, which is committed to the adipocytic lineage and to adipocyte differentiation via intracellular fat droplet accumulation [17]. In the present study, our results demonstrated that the treatment of 3T3-L1 preadipocytes with CG inhibited their differentiation in a dose-dependent manner. CG extract (100 μg/ml) did not cause apparent cytotoxicity in both the undifferentiated or differentiated 3T3-L1 cells. Moreover, the accumulation of lipid droplets in the 3T3-L1 adipocytes was largely reduced by treatment with CG compared with the untreated differentiated cells, and the inhibitory effect was dose dependent. This result is supported by the quantitative analysis of the intracellular lipid content, which revealed that the cells under treatment with 100 μg/ml CG showed 60% reductions in the triglyceride levels in differentiated cells after 6 days, suggesting that CG led to the strong inhibition of adipogenesis and lipid accumulation under these conditions.

Akt is a key enzyme in the insulin signaling pathway in adipocytes, and the insulin-stimulated phosphorylation of Akt plays critical roles in insulin-induced glucose metabolism, glucose transport, and adipocyte differentiation [8,9]. The overexpression of Akt results in increased glucose uptake and adipocyte differentiation, and the inhibition of Akt expression blocked adipocyte differentiation in 3T3-L1 preadipocytes [18]. In the present study, our results demonstrated that CG significantly decreased the phosphorylation of Akt in a concentration-dependent manner in 3T3-L1 adipocytes.

Preadipocyte differentiation is controlled by a subtle balance of serial and interdependent transcription factors [6]. C/EBPδ and C/EBPβ are rapidly and transiently expressed during the early stages of adipocyte differentiation, prior to the transcriptional activation of adipocyte-specific genes [19]. These genes act synergistically to induce the expression of C/EBPα and PPARγ, which promotes the expression of a set of genes involved in adipocyte maturation and differentiation [19]. Therefore, we investigated whether decreased Akt and GSK3β phosphorylation induced the decreased expression of adipogenic genes, such as C/EBP and PPARγ. Our results showed that the CG treatment strongly inhibited the expression of C/EBPβ in a dose-dependent manner in 3T3-L1 adipocytes and that the inhibition of adipocyte differentiation by CG correlates with the inhibition of C/EBPβ expression, which plays a critical role in adipocyte differentiation. Moreover, our data showed that CG significantly attenuated the expression of PPARγ and C/EBPα compared to DMII-induced adipocytes, indicating that CG inhibited adipogenesis in the 3T3-L1 cells by down-regulating PPARγ and C/EBPα expression.

More interestingly, the insulin signaling pathway activates Akt and induces the serine/threonine phosphorylation of the downstream target, GSK3β. Akt appears to participate in the insulin signaling pathway through the phosphorylation of GSK3β and by stimulating GLUT4 translocation [20]. Our data showed that after treatment with CG, the 3T3-L1 adipocytes exhibited marked decreases in the phosphorylation level of GSK3β (Ser-9), suggesting that the decreased phosphorylation of GSK3β at Ser-9 could lead to increased phosphorylation of its corresponding substrates. GSK3β is a ubiquitously expressed serine/threonine protein kinase and had been reported as the C/EBPβ or C/EBPα kinase [13,21]. The insulin-induced dephosphorylation of C/EBPα involved the inactivation of GSK3β, and treatment with the GSK3β inhibitor, lithium, prevented the differentiation of 3T3-L1 preadipocytes into adipocytes [13]. Shim et al. showed that the GSK3β-mediated phosphorylation of C/EBPα targets it for proteasomal degradation [22], and another study also demonstrated that the Fbxw7-dependent degradation of C/EBPα was dependent on the phosphorylation of Thr222 and Thr226, which are GSK3β phosphorylation sites [23]. Furthermore, the Akt signaling cascade is considered important for adipogenesis, as it appears to activate PPARγ and C/EBPα during the induction of 3T3-L1 adipocyte differentiation [24]. Xu et al. showed that the expression of Akt induces an important association between the PI3-kinase-PKB/Akt signal cascade and the transcription factors PPARγ and C/EBPα in the induction of 3T3-L1 adipocyte differentiation [9]. Taken together, our results revealed that the suppression of adipogenesis by CG was caused by the decreased levels of C/EBPβ, C/EBPα, and PPARγ expression through the attenuation of Akt and GSK3β phosphorylation.

The expression of C/EBPα and PPARγ synergistically induces terminal differentiation by transactivating the expression of downstream adipocyte-specific gene, aP2, which is gene involved in the maturation of adipocytes. Therefore, the effect of CG on the expression of aP2 was investigated during 3T3-L1 differentiation. Under differentiation conditions, the expression of aP2 was significantly decreased by CG treatment. Together, our results demonstrated that CG strongly suppressed the expression of critical genes involved in creating and maintaining the adipocyte phenotype and reduced lipid storage and accumulation in 3T3-L1 adipocytes.

## Conclusions

In the present study, we demonstrated that CG inhibited adipocyte differentiation and adipogenesis in 3T3-L1 adipocytes. Our results revealed that CG inhibited adipocyte differentiation by suppressing Akt and GSK3β phosphorylation and their activities, leading to the attenuation of the expression of C/EBPβ, C/EBPα, and PPARγ. Moreover, we showed that CG treatment also decreased the level of aP2 and reduced the lipid accumulation in 3T3-L1 cells. Collectively, these findings provide strong evidence that CG might inhibit adipogenesis through the Akt pathway in 3T3-L1 adipocytes.

## Abbreviations

CG: Centipede grass; PPARγ: Peroxisome proliferating-activated receptor-gamma; C/EBPα: CCAAT-enhancer binding protein-alpha; C/EBPβ: CCAAT-enhancer binding protein-beta; GSK3β: Glycogen synthase kinase-3 beta.

## Competing interests

The authors declare that they have no competing interests.

## Authors’ contributions

HJP, SY, and MKL performed a chemical assay and cell biology studies of cultured cells. BYC, SSL, GMC, SNK, YMS, GSK, and JHC conceived the idea, designed the experiments, and interpreted the experimental results. All authors contributed to manuscript preparations and approved the final manuscript.

## Pre-publication history

The pre-publication history for this paper can be accessed here:

http://www.biomedcentral.com/1472-6882/12/230/prepub
